# Beidseitiges Thoraxtrauma – „double the trouble“?

**DOI:** 10.1007/s00104-023-01891-0

**Published:** 2023-06-02

**Authors:** Katharina Schmelzer, Franziska Ziegenhain, Claudio Canal, Hans-Christoph Pape, Valentin Neuhaus

**Affiliations:** 1 Chirurgische Klinik, Kantonsspital Glarus (KSGL), Burgstr. 99, 8750 Glarus, Schweiz; 2 Klinik für Traumatologie, Universitätsspital Zürich (USZ), Universität Zürich (UZH), Rämistr. 100, 8091 Zürich, Schweiz

**Keywords:** Brustkorbverletzung, Rippenfraktur, Lungenkontusion, Komplikation, Outcome, Chest trauma, Rib fracture, Pulmonary contusion, Complication, Outcome

## Abstract

**Hintergrund:**

Das Thoraxtrauma ist mit einer hohen Morbidität und Mortalität assoziiert. Zur Festlegung der Behandlungsstrategie bei Patienten mit Thoraxtrauma ist eine Abschätzung dieses Risikos elementar.

**Ziel der Arbeit:**

Ziel der Arbeit war 1) eine Analyse der Begleitverletzungen bei ein- bzw. beidseitigen Rippenfrakturen respektive Lungenkontusionen. Des Weiteren erfolgte 2) eine Evaluierung hinsichtlich von Unterschieden in der Komplikationsrate bei ein- bzw. beidseitigen Rippenfrakturen respektive Lungenkontusionen.

**Material und Methoden:**

Retrospektive Analyse aller stationären Patienten mit einem Thoraxtrauma während einer 5‑Jahres-Periode in einem Level-I-Trauma-Zentrum. Wir verwendeten bi- und multivariate Analysen, um die Assoziation von ein- bzw. beidseitigen Rippenfrakturen respektive Lungenkontusionen mit diversen Begleitverletzungen und Outcomes zu ermitteln.

**Ergebnisse:**

Insgesamt 714 Patienten mit mindestens einer Rippenfraktur oder Lungenkontusion wurden analysiert. Patienten mit Lungenkontusionen waren signifikant jünger als Patienten ohne (45 vs. 59 Jahre). Patienten mit beidseitigen Rippenfrakturen oder Lungenkontusionen hatten signifikant häufiger eine zusätzliche Verletzung der Brustwirbelsäule. Der durchschnittliche Injury Severity Score (ISS) lag bei 19.

Komplikationen traten bei 36 % der Patienten auf. Beidseitige Thoraxverletzungen führten bis zu einer 70 %igen Erhöhung der Komplikationsrate. Die Einlage von Thoraxdrainagen, zusätzliche Becken- und Abdominalverletzungen sowie höheres Alter waren signifikante Risikofaktoren für Komplikationen. Die Mortalität lag bei 10 % und war bei beidseitigen Thoraxverletzungen mehr als doppelt so hoch. Höheres Alter, Schädel- und Beckenverletzungen waren Prädiktoren hierfür.

**Diskussion/Schlussfolgerungen:**

Bilaterale Verletzungen wiesen klar höhere Mortalitäts- und Komplikationsraten auf. Die genannten signifikanten Risikofaktoren müssen bei der Behandlung bedacht werden. Beim beidseitigen Thoraxtrauma sollte zudem gezielt nach einer zusätzlichen Brustwirbelsäulenverletzung gesucht werden.

## Abstract (en)

**Background:**

Thoracic trauma is associated with a high morbidity and mortality. Assessing the risk for complications is essential for planning the further treatment strategies and managing resources in thoracic trauma.

**Objective:**

The aim of the study was to analyze concomitant injuries in unilateral and bilateral rib fractures and pulmonary contusions and evaluate differences in complication rates between the two.

**Material and methods:**

In a retrospective study, data from all patients diagnosed with thoracic trauma at a level I trauma center were analyzed. Bivariate and multivariate analysis were used to examine an association of unilateral or bilateral rib fractures, serial rib fractures, and pulmonary contusions with multiple injuries and outcomes. In addition, multivariate regression analysis was utilized to determine the impact of age, gender and additional injuries on outcome.

**Results:**

A total of 714 patients were included in the analysis. The mean Injury Severity Score (ISS) was 19.

Patients with an additional thoracic spine injury had a significantly higher incidence of bilateral rib fractures. Pulmonary contusions were associated with younger age. Abdominal injuries were predictors for bilateral pulmonary contusions. Complications occurred in 36% of the patients. Bilateral injuries increased the complication rate up to 70%. Pelvic and abdominal injuries as well as the need for a chest drain were significant risk factors for complications. The mortality rate was 10%, with higher age, head and pelvic injuries as predictors.

**Conclusion:**

Patients with bilateral chest trauma had an increased incidence of complications and a higher mortality rate. Bilateral injuries and significant risk factors must therefore be considered. Injury of the thoracic spine should be excluded in those patients.

Das Thoraxtrauma ist sowohl auf chirurgischen Notfallstationen als auch im Schockraum eine häufige Entität. Um die Verletzungsschwere sowie die nötigen Ressourcen korrekt abschätzen und einen adäquaten Behandlungsplan aufstellen zu können, sind Kenntnisse über typische assoziierte Verletzungen und eine Einschätzung zur Entwicklung von Komplikationen essenziell.

## Hintergrund und Fragestellung

Thoraxverletzungen machen etwa ein Viertel aller Verletzungen aus und haben damit hohe Bedeutung in der Behandlung traumatologischer Patienten [1]. Typische thorakale Verletzungen sind Rippenfrakturen, Lungenkontusionen, Pneumo-/Hämatothorax und thorakale vaskuläre Verletzungen [2, 3].

Patienten mit Thoraxverletzungen haben in über 50 % Begleitverletzungen [4]. Hierzu zählen unter anderem Verletzungen der Extremitäten oder des Abdomens. Das gleichzeitige Vorliegen thorakaler und extrathorakaler Begleitverletzungen beeinflusst maßgeblich das (zeitliche) Management und erhöht verstärkt die Mortalität und Morbidität [5–7]. Exemplarisch sind Lungenkontusionen bei der Entscheidung, ob Frakturen langer Röhrenknochen primär definitiv versorgt werden, zu erwähnen [5]. So sollte eine Femurschaftfraktur bei Patienten mit Lungenkontusionen nicht primär mittels Reaming und Marknagelosteosynthese versorgt werden, da ein Lungenödem beim Vorliegen von Lungenkontusionen deutlich verstärkt auftreten kann [8].

Thorakale Verletzungen haben zudem einen hohen Einfluss auf den Hospitalisationsverlauf. Van Vledder et al. zeigten in ihrer Studie, dass vier oder mehr frakturierte Rippen bei Patienten über 65 Jahre mit einer erhöhten Pneumonierate sowie Mortalität assoziiert waren. Neben der Anzahl Rippenfrakturen erhöhte auch die Notwendigkeit der Einlage einer Thoraxdrainage sowie das Alter die Pneumonierate und Mortalität [9]. Ein Thoraxtrauma beeinflusst darüber hinaus die Dauer einer invasiven Beatmung und die Aufenthaltsdauer auf der Intensivstation [5, 10].

Um die Ressourcen sinnvoll zu verteilen, ist das Erkennen von Patienten mit einem erhöhten Risiko für Morbidität und Mortalität wichtig. Eine offene Frage ist, ob bestimmte thorakale und Begleitverletzungen das Risiko für Komplikationen und Mortalität erhöhen und ob beidseitige Verletzungen möglicherweise zu einer Verdopplung dieses Risikos führen könnten (auch als „double the trouble“ bezeichnet). Wir wollen mit unserer Studie folgende Fragen beantworten:

Welche Verletzungen sind mit einseitigen bzw. beidseitigen Rippenfrakturen oder Lungenkontusionen assoziiert? Gibt es Unterschiede in der Mortalitäts- und Komplikationsraten zwischen ein- und beidseitigen Thoraxverletzungen und was sind Prädiktoren für ein negatives Outcome?

## Studiendesign und Untersuchungsmethoden

In einer retrospektiven Studie wurden die Daten aller stationären Patienten eines Level-I-Trauma-Zentrums während einer 5‑Jahres-Periode untersucht. Für die Studie liegt ein Ethikvotum vor (PB_2016-01888).

Eingeschlossen wurden schließlich alle Patienten, welche zum Zeitpunkt der Behandlung älter als 17 Jahre waren sowie ein stumpfes Thoraxtrauma mit zumindest CT-graphischer bestätigter Rippenfraktur oder Lungenkontusion erlitten hatten. Das Gesamtkollektiv umfasste 714 Patienten.

Im Rahmen der vorliegenden Studie wurden die Komplikationsrate und die Mortalität während des stationären Verlaufs näher untersucht. Zur Datenerhebung wurden die elektronischen Patientenakten genutzt, die neben klinischen Befunden und Berichten auch Bildgebung und Laborergebnisse enthielten.

Das Ausmaß der Verletzung (Anzahl und Seite der Rippenfrakturen, das Vorliegen einer Lungenkontusion, Hämatothorax und/oder Pneumothorax) wurde durch Analyse der vorliegenden Bildgebung (Computertomographie und/oder Röntgen des Thorax) und der vorliegenden Operationsberichte beurteilt. Die Auswertung aller Bilder erfolgte durch Facharztanwärter der Chirurgie mit Spezialisierung in Traumatologie unter Berücksichtigung der radiologischen Befunde, welche von einem Facharzt für Radiologie erstellt wurden. Die Angaben zu Geschlecht, Alter, Komorbiditäten, weiteren Verletzungen (weitere Unterteilung in Verletzungen des Thorax, des Kopfes, der Extremitäten, der Wirbelsäule, des Beckens und des Abdomens) und Hospitalisationsdauer (inkl. Intensivstunden) sowie Komplikationen (inkl. Mortalität) während der Hospitalisation wurden aus der elektronischen Patientenakte sowie den International Classification of Diseases (ICD, Ausgabe 10) Codes entnommen. Die ICD-Codes wurden von geschulten Ärzten zur DRG-Abrechnung erhoben. Die Abbreviated Injury Scale (AIS) sowie der Injury Severity Score (ISS) wurden ebenfalls erhoben. Die Daten zu Einlage und Anzahl der Thoraxdrainagen wurden aus den codierten Eingriffen der Schweizerischen Operationsklassifikation (CHOP) und den vorliegenden Operationsberichten entnommen. Entsprechend der gängigen Literatur wurde als unerwünschte Folgeerscheinung im Sinne einer Komplikation das Auftreten eines oder mehrerer der folgenden Krankheitsbilder während der Hospitalisation festgelegt: akute Blutungsanämie, Pneumonie, Pleuraempyem, Sepsis, respiratorische Insuffizienz, Intubation, temporäre Tracheotomie, Harnwegsinfektion, thrombembolische Ereignisse (Lungenembolie und tiefe Venenthrombose), disseminierte intravasale Gerinnung, akute Niereninsuffizienz, Herzrhythmusstörungen und akuter Myokardinfarkt [11, 12].

In bi- und multivariaten Analysen wurde die Assoziation von ein- bzw. beidseitigen Rippenfrakturen und Lungenkontusionen mit einem negativen Outcome untersucht. Als Outcomeparameter wurden Tod oder das Auftreten einer Komplikation definiert. Als Kovariaten wurden zudem Alter, Geschlecht, Verletzungen des Kopfes, der Extremitäten, der Wirbelsäule (in Subanalysen wurde die Wirbelsäule noch weiter unterteilt in Hals‑, Brust- und Lendenwirbelsäule), des Abdomens sowie des Beckens in multivariate logistische Regressionsanalysen eingeschlossen. Für die statistische Analyse verwendeten wir IBM SPSS Version 26 (IBM, Armonk, New York, USA). Die statistische Signifikanz wurde auf *p* < 0,001 festgelegt.

## Ergebnisse

### Übersicht

In der 5‑Jahres-Periode wurden 714 Patienten mit einem stumpfen Thoraxtrauma und mindestens einer Rippenfraktur oder Lungenkontusion stationär behandelt. 660 Patienten (92 %) hatten mindestens eine Rippenfraktur. Bei 499 Patienten (70 %) lagen einseitige, bei 161 Patienten (23 %) beidseitige Rippenfrakturen vor. Eine Lungenkontusion lag insgesamt bei 188 Patienten (26 %) vor, davon hatten 96 Patienten (13 %) eine einseitige und 92 (13 %) beidseitige Lungenkontusionen (Tab. 1).

**Table 1:** 

		Total		Rippenfrakturen				Lungenkontusionen					
Parameter		(*n* = 714)		Einseitig (*n* = 499)		Beidseitig (*n* = 161)		Keine (*n* = 526)		Einseitig (*n* = 96)		Beidseitig (*n* = 92)	
		*n*	%	*n*	%	*n*	%	*n*	%	*n*	%	*n*	%
Alter (Jahre ± SD)		55 ± 19	–	55 ± 19	–	59 ± 17	–	59 ± 18	–	46 ± 17	–	44 ± 19	–
Geschlecht	Weiblich	221	31	154	31	53	33	180	34	18	19	23	25
	Männlich	493	69	345	69	108	67	346	66	78	81	69	75
Komorbiditäten vorliegend		377	53	246	49	111	69	300	57	31	32	46	50
AIS Thorax ± SD		2,5 ± 0,9	–	2,3 ± 0,8	–	2,9 ± 1,1	–	2,3 ± 0,9	–	3,0 ± 0,5	–	3,3 ± 0,6	–
Rippenfrakturen	Keine	54	7,6	0	–	0	–	0	–	25	26	29	32
	Einseitig	499	69	499	100	0	–	407	77	57	59	35	38
	Beidseitig	161	23	0	–	161	100	119	23	14	15	28	30
Anzahl frakturierter Rippen (*n* ± SD)		4,7 ± 3,9	–	3,8 ± 2,6	–	9,0 ± 4,6	–	4,8 ± 3,6	–	3,7 ± 3,9	–	5,2 ± 5,6	–
Lungenkontusion		188	26	92	18	42	26	0	–	96	100	92	100
Pneumothorax		212	30	137	28	64	40	120	23	39	41	53	58
Hämatothorax		110	15	63	13	45	28	73	14	14	15	23	25
Fraktur Brustwirbelsäule		136	19	74	15	55	34	84	16	19	20	33	36
Fraktur Klavikula		115	16	78	16	30	19	70	13	26	27	19	21
Fraktur Skapula		95	13	59	12	33	20	56	11	18	19	21	23
Fraktur Sternum		69	10	28	5,6	37	23	44	8,4	8	8,3	17	18
Thoraxdrainagen	Keine	555	78	409	82	98	61	436	83	72	75	47	51
	Einseitig	109	15	76	15	29	18	61	12	21	22	27	29
	Beidseitig	50	7,0	14	2,8	34	21	29	5,5	3	3,1	18	20
ISS ± SD		19 ± 16	–	17 ± 15	–	24 ± 17	–	17 ± 15	–	19 ± 12	–	30 ± 18	–
Verletzungen an den Extremitäten		449	63	299	60	111	69	311	59	71	74	67	73
Verletzungen des Kopfes		446	63	292	59	116	72	315	60	66	71	67	73
Verletzungen an der Wirbelsäule		251	35	146	29	88	55	172	33	35	36	44	48
Abdominalverletzung		198	28	125	25	58	36	130	25	22	23	46	50
Verletzungen am Becken		128	18	79	16	40	25	85	16	15	16	28	30

*AIS* Abbreviated Injury Scale, *ISS* Injury Severity Score, *n* Anzahl, *SD* Standardabweichung

### Ein- und beidseitige Rippenfrakturen

Die Anzahl an frakturierten Rippen betrug bei Verletzungen von nur einem Hemithorax 3,8 (SD ± 2,6), bei Patienten mit beidseitigen Rippenfrakturen 9 (SD ± 4,6). Patienten mit zusätzlichen Wirbelsäulenverletzungen (OR 2,9, 95 %-CI 2,0–4,2, *p* < 0,001) hatten signifikant häufiger beidseitige Rippenfrakturen. Unterteilte man die Verletzungen der Wirbelsäule in Hals‑, Brust- und Lendenwirbelsäulenverletzungen, waren lediglich Verletzungen der Brustwirbelsäule signifikant mit beidseitigen Rippenfrakturen assoziiert. Thoraxdrainagen wurden bei beidseitigen Rippenfrakturen doppelt so häufig gelegt wie bei einseitigen Verletzungen.

In einer Subanalyse hatten ältere Patienten gehäuft Rippenserienfrakturen (3 oder mehr benachbarte Rippen frakturiert; OR 1,01, 95 %-CI 1,002–1,02, *p* = 0,014). Es lagen deutlich weniger Lungenkontusionen in der Gruppe mit Rippenserienfrakturen vor (22 % vs. 34,6 %), andere thorakale Verletzungen (Pneumothorax, Hämatothorax, Frakturen der Brustwirbelsäule oder Skapula) jedoch mehr als doppelt so häufig im Vergleich zu Patienten ohne Rippenserienfrakturen.

### Ein- und beidseitige Lungenkontusionen

Junges Patientenalter war signifikant mit dem Vorliegen von Lungenkontusionen assoziiert (OR 0,96 für 1 Jahr älter, 95 %-CI 0,95–0,97, *p* < 0,001). Das gleichzeitige Vorliegen einer Abdominalverletzung war ein Prädiktor für beidseitige Lungenkontusionen (OR 3,6, 95 %-CI 1,9–6,9, *p* < 0,001).

### Outcome

Insgesamt entwickelten 259 Patienten (36 %) Komplikationen (Tab. 2 und 3). Komplikationen kamen deutlich häufiger bei beidseitigen Verletzungen vor (48 % bei beidseitigen vs. 33 % bei einseitigen Rippenfrakturen, 48 % bei beidseitigen vs. 29 % bei einseitigen Lungenkontusionen). Parallel dazu sehen wir eine Komplikationsrate von 20 % (einseitige Verletzungen) bis 32 % (beidseitige Verletzungen) bei Leichtverletzten (ISS < 17), welche auf 34 % (einseitige Verletzungen) bis 59 % (beidseitige Verletzungen) ansteigt bei polytraumatisierten Patienten (ISS ≥ 17). Signifikante Risikofaktoren waren die Notwendigkeit der Einlage von 1 oder 2 Thoraxdrainagen sowie Verletzungen am Becken, des Abdomens, der Wirbelsäule sowie das höhere Alter (Tab. 4).

**Table 2:** 

	Total		Rippenfrakturen				Lungenkontusionen					
	(*n* = 714)		Einseitig (*n* = 499)		Beidseitig (*n* = 161)		Keine (*n* = 526)		Einseitig (*n* = 96)		Beidseitig (*n* = 92)	
Verweildauer (Tage ± SD)	11 ± 14		9,8 ± 11		16 ± 22		10 ± 14		11 ± 13		15 ± 17	
Intensivstunden (Stunden ± SD)	76 ± 182		58 ± 168		131 ± 223		54 ± 118		93 ± 280		181 ± 286	
Beatmungsstunden (Stunden ± SD)	39 ± 109		27 ± 96		74 ± 142		26 ± 75		46 ± 156		106 ± 172	
Verstorben (*n*, %)	69	10 %	46	7,2 %	30	19 %	46	8,7 %	6	6,3 %	17	18 %
Komplikationen (*n*, %)	259	36 %	162	33 %	78	48 %	188	36 %	27	28 %	44	48 %

*n* Anzahl, *SD* Standardabweichung

**Table 3:** 

	Total		Rippenfrakturen				Lungenkontusionen					
	(*n* = 714)		Einseitig (*n* = 499)		Beidseitig (*n* = 161)		Keine (*n* = 526)		Einseitig (*n* = 96)		Beidseitig (*n* = 92)	
	*n*	%	*n*	%	*n*	%	*n*	%	*n*	%	*n*	%
Akute Blutungsanämie	98	14	59	12	30	19	70	13	7	7,3	21	23
Intubation	81	11	39	7,8	32	20	54	10	9	9,4	18	20
Herzrhythmusstörungen	65	9,1	40	8,1	23	14	51	9,7	3	3,2	11	12
Pneumonie	51	7,1	27	5,4	19	12	30	5,7	7	7,3	14	15
Sepsis	40	5,6	17	3,4	19	12	20	3,8	9	9,4	11	12
Respiratorische Insuffizienz	34	4,8	19	3,8	13	8,1	19	3,6	4	4,2	11	12
Harnwegsinfektion	34	4,8	23	4,6	10	6,2	28	5,3	3	3,1	3	3,3
Tracheotomie, temporär	32	4,5	14	2,8	13	8,1	13	2,5	4	4,2	15	16
Akute Niereninsuffizienz	25	3,5	7	1,4	17	11	12	2,3	4	4,2	9	9,8
Tiefe Venenthrombose	13	1,8	9	1,8	3	1,9	7	1,3	3	3,1	3	3,3
Lungenembolie	8	1,1	5	1,0	3	1,9	5	1,0	2	2,1	1	1,1
Disseminierte intravasale Gerinnung	7	1,0	3	0,61	4	2,5	5	1,0	1	1,0	1	1,1
Frischer Myokardinfarkt	5	0,70	2	0,40	2	1,2	4	0,76	0	–	1	1,1
Empyem	2	0,28	1	0,20	1	0,62	1	0,19	1	1,0	0	–

*n* Anzahl

**Table 4:** 

	*p*-Wert	Odds Ratio	95 %-CI	
			Untere	Obere
Thoraxdrainagen beidseitig vs. keine	0,001	3,3	1,6	7,0
Thoraxdrainagen einseitig vs. keine	<0,001	2,7	1,7	4,2
Verletzungen am Becken	0,001	2,3	1,4	3,6
Abdominalverletzung	<0,001	2,2	1,4	3,4
Verletzungen an der Wirbelsäule	0,001	1,9	1,3	2,8
Alter	<0,001	1,0	1,01	1,03

*CI* Konfidenzintervall mit jeweils unterer und oberer Grenze, *Odds Ratio* Chancenverhältnis

Die Hospitalisationsdauer war bei beidseitigen Verletzungen (Rippenfrakturen und Lungenkontusionen) durchschnittlich mindestens 4 Tage länger als bei einseitiger Verletzung. Bei den Intensivstunden und Beatmungsstunden zeigte sich eine mehr als doppelt so lange Verweildauer auf der Intensivstation und notwendige Beatmungsstunden bei beidseitigen Verletzungen.

Die Mortalität betrug 10 % (*n* = 69) und war bei beidseitigen Verletzungen mehr als doppelt so hoch (einseitig 7 % vs. beidseitig 19 % bei Rippenfrakturen, einseitig 6 % vs. beidseitig 18 % bei Lungenkontusionen). Signifikante Prädiktoren für das Versterben waren Verletzungen am Kopf (OR 3,2, *p* = 0,001) sowie des Beckens (OR 2,9, *p* < 0,001) und das höhere Alter (OR 1,03, *p* < 0,001). In Analogie dazu sehen wir eine Mortalität von 1,6 % (einseitige Verletzungen) bis 6,3 % (beidseitige Verletzungen) bei Leichtverletzten (ISS < 17), welche auf 14,3 % (einseitige Verletzungen) bis 27 % (beidseitige Verletzungen) ansteigt bei polytraumatisierten Patienten (ISS ≥ 17).

Nach Aufschlüsselung der einzelnen Rippenfrakturen bez. der Höhe und der damit korrelierenden Mortalitäts- und Komplikationsrate, zeigte sich trendmäßig eine zunehmende Komplikationsrate von apikal nach kaudal (mit Ausnahme der 1. Rippe), wobei die Komplikationsrate rechts gegenüber links leicht höher war. Die Mortalität zeigte hingegen eine abnehmende Rate von apikal nach kaudal, wobei die Mortalitätsrate links gegenüber rechts leicht höher war (Tab. 5).

**Table 5:** 

Links			Rippe	Rechts		
Komplikationen (%)	Mortalität (%)	Anzahl		Anzahl	Mortalität (%)	Komplikationen (%)
47	19	79	Rippe 1	73	19	51
40	20	119	Rippe 2	114	17	40
40	19	183	Rippe 3	150	16	42
42	16	206	Rippe 4	199	17	43
43	15	249	Rippe 5	204	15	43
41	15	241	Rippe 6	197	14	44
40	15	218	Rippe 7	182	13	45
41	14	167	Rippe 8	140	13	47
42	14	139	Rippe 9	107	14	54
41	16	102	Rippe 10	90	12	61
49	18	67	Rippe 11	68	9	56
45	16	38	Rippe 12	36	5,6	61

## Diskussion

Das Ziel dieser Studie war, zu untersuchen, welche Faktoren mit ein- oder beidseitigen Rippenfrakturen oder Lungenkontusionen assoziiert sind. Gleichzeitig wollten wir prüfen, inwiefern die Mortalität und das Risiko für Komplikationen davon abhängen, ob ein- oder beidseitige Rippenfrakturen oder Lungenkontusionen vorliegen.

Unsere Ergebnisse zeigten, dass das Alter der Patienten einen wichtigen Einfluss auf das Ausmaß der thorakalen Verletzungen hat. Ältere Patienten hatten eher beidseitige als einseitige Verletzungen sowie eher Rippenfrakturen als Lungenkontusionen. Der Einfluss des Alters auf das Ausmaß der Rippenfrakturen oder Lungenkontusionen ist nachvollziehbar. Eine Begründung hierfür könnte in der zunehmenden Fragilität bzw. abnehmenden Elastizität der Knochen im Alter liegen. In der Konsequenz kann postuliert werden, dass es in der mittleren Altersgruppe eher zu Lungenkontusionen und weniger rasch als bei den älteren Patienten zu Frakturen kommt. Agnew et al. untersuchten in ihrer 2015 veröffentlichen Studie die strukturelle Festigkeit der Rippenknochen bezogen auf das Alter der Patienten. Hierbei zeigte sich bei der für eine Rippenfraktur notwendigen aufgewendeten Kraft ein Peak bei Erwachsenen zwischen 25 und 40 Jahren. Parallel zu diesen Ergebnissen konnte eine lineare Abnahme der strukturellen Festigkeit mit zunehmendem Alter nachgewiesen werden [13].

Beim beidseitigen Thoraxtrauma muss gemäß unseren Daten aktiv nach einer Brustwirbelsäulenverletzung gesucht werden. Diese Beobachtung deckt sich mit Daten aus anderen Studien [14]. Verletzungen der Brustwirbelsäule treten generell bei 3 % der Patienten mit stumpfen Traumata auf [15]. In einer Studie von Schulz et al. zu knöchernen Verletzungen bei Polytraumata zeigt sich eine Fraktur im Bereich des thorakolumbalen Übergangs in Kombination mit Rippenbrüchen bei 39,1 % der Patienten. In einer weiteren Studie finden sich bei 48,6 % der Patienten mit einem schweren Thoraxtrauma Frakturen des thorakolumbalen Übergangs [16].

Beidseitige Rippenfrakturen oder Lungenkontusionen erhöhten die Mortalität um mehr als das Doppelte und die Komplikationen um mehr als 50 %. Patienten mit beidseitigen Thoraxverletzungen waren in unserer Studie mehr als doppelt so lange auf der Intensivstation und mussten mehr als doppelt so lange beatmet werden. Die Rate an Thoraxdrainageneinlagen war ebenfalls mehr als doppelt so hoch, was wiederum das Risiko an Komplikationen deutlich erhöhte. Es fällt zudem auf, dass thoraxspezifische Komplikationen wie Pneumonie, respiratorische Insuffizienz, Intubation oder Tracheotomie doppelt so häufig vorkommen. Frühere Studien fanden ebenfalls diesen Zusammenhang [17–19].

Eine Aussage darüber, ob die Beidseitigkeit von Verletzungen sowie die Thoraxdrainageneinlage per se Prädiktoren für die Entwicklung von Komplikationen oder einfach nur als Zeichen der Schwere des Traumas zu werten sind, bleibt schwierig. Van Vledder et al. beschreiben in ihrer Studie eine signifikant erhöhte Mortalität während der Hospitalisation sowie ein signifikant erhöhtes Risiko für die Entwicklung einer Pneumonie nach der Notwendigkeit der Einlage einer Thoraxdrainage [9]. Dagegen spricht eine 2010 veröffentlichte Studie, die als einzigen signifikanten Risikofaktor für die Entwicklung einer Pneumonie nach schwerem Thoraxtrauma die präklinische Intubation und Beatmung von Patienten feststellte. Eine Aspiration, Lungenkontusionen und das Vorhandensein eines Hämatothorax zeigten ebenfalls ein erhöhtes, aber nicht signifikantes Risiko für die Entwicklung einer Pneumonie in einer weiteren Studie [20]. Unsere Daten lassen diesbezüglich keine abschließende Beurteilung zu.

Das zunehmende Alter hat zudem einen negativen Einfluss auf die Komplikations- und Mortalitätsrate, wie bereits in mehreren Studien nachgewiesen [21–23]. Ein klarer Cut-off bez. einer Altersgrenze besteht nicht. Ab 45 Jahren scheint sich eine Erhöhung der Mortalität abzuzeichnen [21, 24, 25], dies obwohl bei Patienten > 65 Jahren vermehrt Monotraumata des Thorax aufgrund niedrigkinetischer Unfälle vorliegen [7, 26]. Die Mortalität scheint in dieser Altersgruppe somit nicht hauptsächlich durch die Schwere der Begleitverletzungen bedingt zu sein.

Mehrere Studien zeigten, dass besonders ältere Patienten mit Thoraxtrauma signifikant häufiger eine Pneumonie entwickelten [7, 27]. Dies wiederum erhöhte die Mortalität exponentiell [7, 22, 27]. In unserer Kohorte beobachteten wir ebenfalls eine etwa doppelt so hohe Rate an Pneumonien bei Patienten über 65 Jahren mit einer entsprechend hohen Mortalität.

Es handelt sich um eine retrospektive Datenerhebung. Um eine möglichst einheitliche Erfassung der Daten sowie Reduktion des Bias bezüglich Rippenfrakturen und Lungenkontusionen zu gewährleisten, wurden diese von wenigen hierzu geschulten Facharztanwärtern erhoben. Bei insgesamt 19 Fällen lag kein Computertomogramm des Thorax vor, die Abweichungen entstanden unter anderem durch Versterben der Patienten vor der (Verlaufs‑)Bildgebung. Dies könnte zu kleineren Abweichungen bez. der Gesamtzahl an Lungenkontusionen sowie Rippenfrakturen führen, da bei alleiniger nativradiologischer Initial- und Verlaufsdiagnostik nach 24 h eine Lungenkontusion ggf. noch nicht abgrenzbar ist und Rippenfrakturen schwieriger zu diagnostizieren sind [28–32].

## Schlussfolgerung: „There is (no) double the trouble“

Die Aussage „double the trouble“ trifft bezüglich des beidseitigen Thoraxtraumas meist zu. Einige untersuchte Parameter, wie die Verweildauer auf der Intensivstation, Dauer der Beatmung, die Mortalitätsrate, das Auftreten pulmonaler Komplikationen sowie die Notwendigkeit zur Einlage einer Thoraxdrainage waren in den von uns erhobenen Daten bei beidseitigen Verletzungen mehr als doppelt so hoch. An anderer Stelle ließ sich dieser Schluss nicht ziehen (allgemeine Komplikationsrate sowie Verweildauer). Das Alter hat einen wichtigen Einfluss auf die Verletzungsschwere sowie auch das Auftreten von Komplikationen bis hin zu Tod.

Dies ist eine für die Planung der weiteren Betreuung oder Versorgung mehrfach verletzter Patienten oder auch älterer Patienten relevante Information, die für Unfallchirurgen in der Entscheidungsfindung bez. des weiteren Vorgehens hilfreich ist. Die Kenntnis über assoziierte Verletzungen kann im klinischen Alltag eine gezielte Suche nach zusätzlichen Verletzungen beschleunigen.

## Fazit für die Praxis

Das beidseitige Thoraxtrauma erhöht die allgemeine Komplikationsrate um 50 %. Eine beidseitige Verletzung bedeutet eine Verdoppelung der Intensiv- und Beatmungszeit, eine Verdoppelung spezifischer Komplikationen (Pneumonie, respiratorische Insuffizienz, Intubation, Tracheotomie) und der Mortalität. Um in der Praxis die Risiken rasch einordnen und in die Behandlungsstrategie einbeziehen zu können, sollten Risikofaktoren (z. B. Einlage von Thoraxdrainagen, Becken- und Abdominalverletzungen, hohes Alter) in die Planung miteinbezogen und gezielt nach assoziierten Verletzungen (z. B. Brustwirbelsäulenverletzungen) gesucht werden.
